# Captivity drives multi-generational shifts in the gut microbiome that mirror changing animal fitness

**DOI:** 10.1128/mbio.03516-25

**Published:** 2026-01-23

**Authors:** Candace L. Williams, Claire E. Williams, Shauna N. D. King, Debra M. Shier

**Affiliations:** 1 Conservation Science, San Diego Zoo Wildlife Alliance 196338 , Escondido, California, USA; 2 University of Nevada 6851, Reno, Nevada, USA; University of California Irvine, Irvine, California, USA

**Keywords:** gut microbiome, host–microbiome interactions, multi-generational sampling, captive breeding, wildlife conservation

## Abstract

Gut microbiomes can dramatically affect host health and fitness, but can shift rapidly under changing environmental conditions. Understanding the interplay between microbiota, environmental pressures, and host responses is critical for predicting species’ resilience, particularly in populations transitioning from the wild to human care for conservation breeding. Although captivity can profoundly alter microbial communities and host physiology, the dynamics of these transitions across generations remain poorly understood. We evaluated gut microbiota and fitness metrics in the endangered Pacific pocket mouse (*Perognathus longimembris pacificus*) during the establishment of a conservation breeding and reintroduction program, spanning five generations. Microbiome composition shifted gradually, stabilizing into a distinct captivity-associated state after two to three generations. These transitions paralleled changes in host weight and reproductive performance, suggesting coordinated host–microbiome adaptation. In addition, we identified microbial taxa correlated with successful reproduction, highlighting potential microbial markers of fitness. Our findings provide the first characterization of gut microbiota in Pacific pocket mice and demonstrate how captivity shapes host–microbiome systems across generations. More broadly, they underscore the importance of considering microbiome dynamics in conservation management and suggest that microbial responses to environmental change may require multiple generations to reach a new stable state.

**IMPORTANCE:**

In human-altered landscapes, animals face numerous threats to their survival, yet little is known about how rapid environmental change affects host–microbiome dynamics across generations. Microbial communities play critical roles in host nutrition, immunity, and overall fitness, and shifts in composition may alter an organism’s ability to adapt. We examined the gut microbiota of the endangered Pacific pocket mouse during the transition from wild to captive environments and across four descendant generations. We found that the microbiome did not immediately shift with captivity but instead stabilized into a distinct, captivity-associated state only after several generations. This study provides the first characterization of gut microbiota in pocket mice and is the first to show, at this resolution, how a wildlife species’ microbiome adapts to environmental change while tracking health and fitness across generations. Our findings highlight the need to incorporate microbiome dynamics into conservation breeding and management strategies.

## INTRODUCTION

Human-induced rapid environmental change threatens native species through habitat loss, fragmentation, and degradation (1; see reviews in references 2–5). Animals that live within these ecosystems must disperse, acclimate, or adapt to persist, but for many species, their options are limited. Extensive habitat loss and/or fragmentation may prevent dispersal, and acclimatization can be constrained by the type and magnitude of environmental perturbations or inherent characteristics or species-specific traits (e.g., dispersal capacity, genetic diversity). Given the rapid nature of these environmental changes, many animals cannot evolve quickly enough to adapt, making human intervention essential for their survival. To prevent imminent extinction, individuals are brought into human care, propagated, and their offspring released into suitable wild habitats (6). However, selective pressures differ between wild and captive environments (7, 8), and adaptations to captivity can prove maladaptive when animals are reintroduced into the wild (9).

Host-associated microbiota play many vital roles in host physiology and may help hosts navigate such environmental change (10–13). Since microbiomes are highly dynamic, they can change on a more rapid timescale than their animal hosts in response to environmental pressures, possibly facilitating microbiome-mediated plasticity (14–22). Alternatively, environmentally driven changes in the gut microbiome may decouple a host from suitable microbiota and lead to negative effects on host health and fitness. Specifically, captivity alters the gut microbiome, resulting in shifts in composition (23). These changes have been associated with detrimental effects on host health, including gastrointestinal disease and metabolic disorders (24–31). The cumulative effects of captivity on both the host and its microbial community likely shape animal health, survival, and ultimately the success of conservation breeding programs.

Despite its importance, the dynamics of host–microbiome transitions between the wild and captivity remain poorly understood. Understanding these processes may be critical for predicting and managing host fitness (32). While the gut microbiome has been identified as a key monitoring indicator for reintroductions in giant pandas (33), to date, conservation actions using microbial augmentation have only been described for soils and amphibian skin, and no publications have described how wildlife and their microbiomes change across generations in captivity (34). Detailed characterization of these microbiome dynamics and how they affect animal health over time would enable management decisions and interventions targeted at the gut microbiome (32, 33, 35–37), which may lead to improved success of conservation breeding programs, reintroductions, and ultimately the recovery of endangered species.

Most studies that evaluate the effect of captivity on animal gut microbiomes have been cross-sectional in design, sampling only established populations in captivity and those in the wild or monitoring a transition over a short time scale (38). Here, we evaluated how environmental change altered the gut microbiome and associated animal fitness in the endangered Pacific pocket mouse (*Perognathus longimembris pacificus*) during a transition to captivity across multiple generations. The Pacific pocket mouse is a heteromyid that was previously thought to be extinct in the 1970s. Small populations were rediscovered in 1993, and the species was emergency listed by the United States Fish and Wildlife Service as endangered (39). Due to the precarious status of the three extant populations, a conservation breeding and reintroduction program was initiated in 2012 with the goal to create three new populations (40). Using 16S rRNA amplicon sequencing in combination with detailed monitoring of the health and reproductive status of pocket mice, we observed the dynamics of simultaneous host and microbial responses as founding individuals transitioned to captivity. We also evaluated these same metrics in descendant matrilineal generations maintained in captivity. By combining both bacterial and host measures, we aimed to better understand the bidirectional interaction between the microbiome and pocket mice undergoing rapid environmental change. We tested the hypothesis that the gut microbiome would change as animals adapted to the *ex situ* environment, and that these changes would persist through multiple generations. Furthermore, we predicted that changes in the gut microbiome elicited by the transition to captivity would correspond to changes in pocket mouse health and fitness across multiple generations.

## MATERIALS AND METHODS

All analyses and associated sample metadata described below can be found in our GitHub repository (https://github.com/clw224/2023-Williams-PPM-WildtoCaptive/), as well as a visual description of our workflow in Fig. S1. All samples and fitness data were collected prior to microbiome hypothesis generation; therefore, we used our sample and data archive to conduct analyses.

### Sample collection

**Fig 1:**
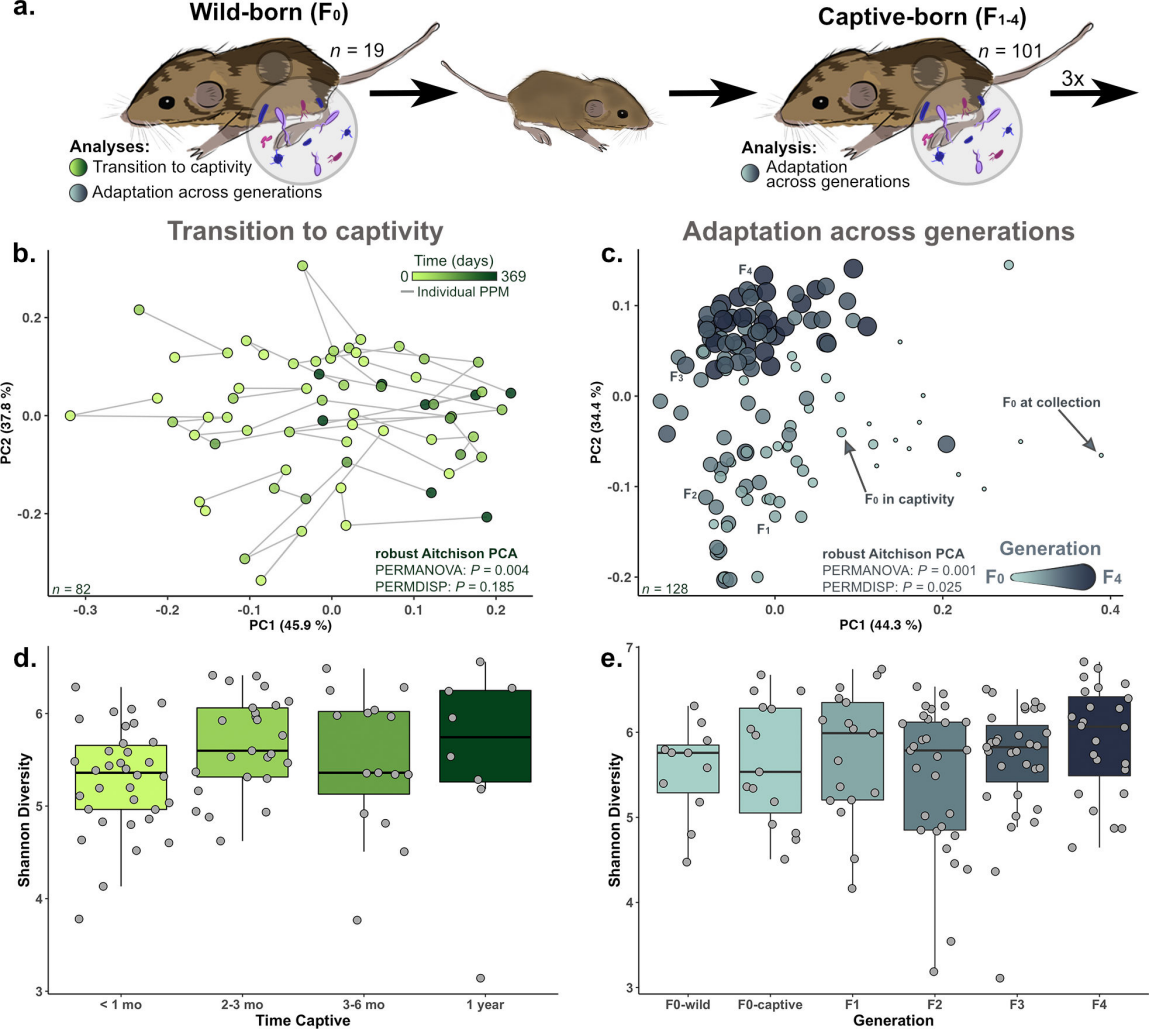
Captivity significantly alters microbiome composition across multiple generations, but not alpha diversity. (**a**) An overview of our experimental design, sampling from both wild-born (F_0_) and captive-born (F_1–4_) individuals in the conservation breeding program. A robust Aitchison principal component analysis (**b**) shows that microbiome composition shifted as F_0_ individuals transitioned to captivity, while Shannon diversity (**d**) remained relatively constant. Community composition showed further changes across generations (**c**) while Shannon diversity did not (**e**).

We leveraged a long-term data set from a conservation breeding and reintroduction program for the endangered Pacific pocket mouse (see Fig. 1a for study overview), consisting of *in situ* and *ex situ fecal* samples. When F_0_ individuals were collected in the wild to establish the program, initial fecal samples (F_0_-wild, *n =* 11) were collected *in situ* either from the trap or directly from the pocket mouse during handling. We inspected traps between animals to ensure the fecal pellets removed from the trap belonged to the target animal and used sterilized tweezers to transfer feces into a vial. For direct samples, we opportunistically collected the feces straight into a vial, as pocket mice often defecate when being handled. After individuals were moved to the breeding facility, *ex situ* samples were collected systematically throughout the first 369 days in captivity in the same manner, except indirect samples were collected from the individual’s enclosure, and direct samples were obtained during a scheduled handling event. For some individuals, we were able to collect repeated samples (Fig. S2). For matrilineal generations F_1+_, one sample was randomly selected for each individual post-weaning, taking into account sex to ensure even sampling across the population (F_1_, *n =* 17; F_2_, *n =* 29; F_3_, *n =* 31; F_4_, *n =* 24). We stored all samples at −20°C prior to analysis.

### Animal fitness data collection

To evaluate fitness metrics *in situ* and *ex situ*, we collected data on weights and reproductive readiness across the entire captive breeding population (F_0_ through F_6_). In pocket mice, reproductive output is limited by females. Females typically cycle frequently throughout the breeding season (from approximately February to September) and can have up to three litters in a single breeding season (41). Reproductive fitness appears to be limited by reproductive readiness by females (estrous cycling) and males (testes position) as much as other metrics such as age or history (42). Here, we use reproductive readiness and successful reproduction as proxies for reproductive fitness. In 2007 and 2008, we conducted trapping *in situ* five nights each week after pocket mice emerged from hibernation (February or March), through the active season (~September) each year. We marked pocket mice for individual identification using Visible Implant Elastomer (Northwest Marine Technology, Inc., Shaw Island, WA, USA) injected in unique color combinations just under the skin on the sides of the tail base. During processing, we weighed each individual and assessed reproductive status. For females, we evaluated reproductive status by visually evaluating vulvar swelling. We rated the degree of vulvar swelling on a four-point scale (1 = not swollen, 4 = maximally swollen) developed for kangaroo rats (43) and adapted for pocket mice (42). For males, we categorized reproductive status using testis position (44, 45) (i.e*.,* 0 = non-scrotal, testes receded into abdomen, not visible; 1 = partially scrotal, testes partially descended into the scrotum; or 2 = scrotal, testes fully descended into the scrotum) (42, 46). We used the same methods to collect fitness metrics *ex situ,* except that for pocket mice housed in the *ex situ* population, we obtained weekly weights for all pocket mice throughout the year. We assessed reproductive status for all females at least every 3 days during the active season. If females had a swelling of ≥2, we monitored them as often as every hour to determine physical estrus (47). We used a swelling score of ≥3, perforation, or the presence of a plug to indicate that an estrous cycle had occurred. We assessed male reproductive condition weekly. To evaluate parentage, we tracked pocket mouse pedigrees through the studbook in PopLink (48) and used PMx to manage breedings (49).

### DNA extraction

We conducted a dual-extraction protocol with solvent extraction for metabolites for a separate, unpublished data set, followed by DNA extraction for bacterial DNA (50). We lyophilized fecal samples (*n =* 184) and homogenized ~3 mg of fecal pellets in 80% methanol in water to extract metabolites (both Optima LC-MS grade, Fisher Chemical). Following metabolite extraction, we used the remaining cell pellet for genomic DNA extraction. In addition to samples, each 96-well plate contained both negative and positive controls for DNA extraction and PCR (ZymoBIOMICS Microbial Community Standard and ZymoBIOMICS Microbial Community DNA Standard, respectively, Zymo Research, Irvine, CA, USA), with samples and controls randomized across both extraction plates. We mechanically lysed samples by two bead-beating steps (5 min), with a heat treatment (65°C, 10 min) in between, and the lysate was further extracted using a Zymo Fecal, Soil, Microbe 96 Magbead kit (Zymo Research, Irvine, CA, USA) on an OT-2 liquid handling robot (Opentrons, Queens, NY, USA) using a protocol modified from Sanders et al. (51). We quantified the resulting genomic DNA by Quant-iT Assay (Invitrogen, Waltham, MA, USA).

### Sequencing library preparation

We prepared sequencing libraries using a protocol modified from Kozich et al. (52) and Williams et al. (30). Briefly, we PCR amplified the V4 region of the 16S rRNA gene using a forward primer (V4f: TATGGTAATTGTGTGCCAGCMGCCGCGGTAA) and reverse primer (V4r: AGTCAGTCAGCCGGACTACHVGGGTWTCTAAT) in a 25-μL reaction mixture with 1× KAPA HiFi Hot Start Ready mix (Kapa Biosystems, Wilmington, MA, USA), 0.2 μM of each primer was added, and sample concentrations ranged between 0.5 and 10 ng genomic DNA. Amplification conditions were as follows: 95°C for 2 min, followed by 25 cycles of 95°C for 20 s, 55°C for 15 s, and 72°C for 30 s, and a final 10-min extension at 72°C. We purified PCR products via gel extraction (Zymo Gel DNA recovery kit; Zymo Research, Irvine, CA, USA) using a 1.0% low-melt agarose gel (National Diagnostics, Atlanta, GA, USA). While the negative controls produced no band, we excised the expected area. We quantified purified PCR products by Quant-iT assay (Invitrogen, Waltham, MA, USA). We combined all samples to yield an equimolar 2 nM pool. Following the manufacturer’s protocol, we sequenced using an Illumina MiSeq reagent kit v2 (500 cycles) (Illumina, San Diego, CA, USA) with the final library concentration of 3.5 pM with 20% Phi-X spiked in.

### 16S rRNA sequence processing

We used QIIME2 and R as described previously for sequence analysis (53). In brief, we imported demultiplexed FASTQ files into QIIME2 (54) and denoised (trimmed and filtered) using the DADA2 plug-in, with the command “denoise-paired” to generate amplicon sequence variants (ASVs) (55). We built a phylogenetic tree by alignment using the MAFFT plug-in, masking using the FastTree plug-in, and rooting it via the phylogeny midpoint plug-in (56, 57). We assigned taxonomy using the feature-classifier “classify-sklearn” with the SILVA full-length 16S database (reference database release 138) (58). Although the negative controls had few reads (<100), we decontaminated the ASV feature table in R via the decontam package (59), and we filtered any ASVs that were not gut-associated from the feature table in QIIME2. We also removed sequences assigned to mitochondria, chloroplasts, or those that lacked phylum-level classifications to avoid issues associated with misamplification.

### Microbiome alpha diversity, beta diversity, core community, and differential abundance analyses

Our statistical analyses of microbiome data fell into two comparisons: (i) determining the changes within a wild-caught individual as they adapted to captivity (F_0_ only), and (ii) evaluating the change in the gut microbiome across generations of mice, from F_0_ through F_4_. To evaluate the transition of bacterial alpha diversity as pocket mice adapted to captivity, we calculated Shannon diversity and Faith’s phylogenetic diversity (PD) following rarefaction (8,500 reads, see rarefaction curves Fig. S3) using QIIME2 and constructed a linear mixed effects model with “days captive,” “year,” and “sex” as fixed effects and individual mouse identifier (“mouse ID”) as a random effect (60–62). “Year” was not found to significantly affect diversity and thus was removed from the model. To quantify compositional beta diversity and determine if it varied across the transition to captivity (days 0–369), we performed a robust Aitchison principal component analysis on the ASV table implemented by the DEICODE plugin in QIIME2 (63). We used the DEICODE distance matrix and measured differences in composition and dispersion (variance) based on the fixed effects of “days captive,” “year,” and “sex” with individual “mouse ID” as a random effect using permutational analysis of variance (PERMANOVA) (64) and betadisper implemented by vegan::adonis and vegan::betadisper*,* respectively, in R (65). We conducted multiple comparisons post hoc after finding significant main effects (i.e., for “days captive” but not “sex” or “year”). We also examined the relationship between the first principal component (PC1) and time in captivity using a Pearson correlation (stats::cor) (66).

We followed a similar approach for cross-generational analyses. To test for differences in Shannon diversity and Faith’s PD across generations, we used a simple linear regression with “generation,” “year,” and “sex” as fixed effects. “Year” and “sex” were not found to be significant and thus were removed from the models. We found that when analyzing changes across generations, significant differences in beta diversity variance emerged (*P =* 0.002, via PERMDISP). To accurately quantify differences in bacterial composition, for generation-based analyses, we randomly trimmed our data set to provide a balance of sample observations across generations (see Table S1) based on several criteria: (i) F_0_ populations, samples were classified as either F_0_-wild (samples taken at animal collection *in situ*), F_0_-captive (following the 3–6 month transition period *ex situ*), (ii) if duplicate samples existed for individuals within these time periods, they were collapsed using the mean ceiling of feature abundances to create one sample per individual, (iii) we randomly selected individuals across generations, but took into account sex, to ensure even sampling across males and females. We then used this collapsed data set to analyze differences in beta-diversity across generations. As described, we used the DEICODE distance matrix and measured differences in composition and dispersion (variance) based on the fixed effects of “generation,” “year,” and “sex” using PERMANOVA (64) and betadisper implemented by vegan::adonis *and* vegan::betadisper*,* respectively, in R (65). We conducted multiple comparisons post hoc after finding significant main effects (i.e., for “generation” but not “sex” or “year”). We also examined the relationship between the first principal component (PC1) and time in captivity using a Pearson correlation (stats::cor) (66).

To determine which ASVs or bacterial families comprised a stable, “core” community that was maintained across generations, we used an abundance-occupancy approach (67). Briefly, ASVs and their families were ranked based on their occupancy and abundance in each generation. We used a less than 2.0% contribution to Bray-Curtis community composition as a cutoff, as suggested by Shade and Stopnisek (67), and Bray-Curtis similarity in our data set plateaued shortly after this cutoff (68). Therefore, we likely included the majority of ASVs that are important to structuring the community beta diversity, thus identifying the taxa that are maintained over generations and are therefore considered potentially important for the community structure of the gut microbiome. To identify which taxa (genera and ASVs) were differentially abundant across generations, we used Maaslin2 (69) in R with total sum scaling-normalization and log-transformation to implement linear models to assess differences in relative abundance of ASVs and genera across generations with a Benjamini-Hochberg multiple comparison correction (70), as it provides a good balance between discovery of statistically significant ASVs and limitation of false positive occurrences.

### Host fitness analyses

To test for effects of generations in captivity on pocket mouse fitness, we used weight, estrous cycles, or testes score as a response variable and generation as a fixed effect, and compared changes in these reproductive measures by generation using Kruskal-Wallis tests due to lack of normality (66, 71). For all outcome variables, we calculated a mean for each individual across their first full breeding season (either the year following their capture or their birth in captivity). For analysis of estrous cycles, we excluded data from time periods when individual pocket mice experienced conditions likely to confound cycle patterns (e.g*.*, pregnancy or postpartum). To characterize reproductive success, we used the package purgeR on pedigrees generated from the studbook (including individuals from generations F_0_–F_6_). To test for relationships between specific microbial taxa on pocket mice reproductive success, we constructed separate generalized linear models implemented with the package in Maaslin2 (69). We rarefied our ASV table to 8500 reads in QIIME2 (54), and then we used pocket mouse reproduction (i.e., whether or not mice reproduced) in their first breeding season (0/1) as a binomial response variable and relative microbial abundance as a fixed effect, correcting for multiple comparisons using a Benjamini-Hochberg approach (70).

## RESULTS

### Captivity rapidly alters microbiome composition

To understand how the gut microbiome changes during the transition to and throughout subsequent generations in captivity, we evaluated the community composition and richness of the gut microbiome across these periods (Fig. 1a). We found that the pocket mouse gut microbiome’s community composition changed rapidly as wild-collected individuals transitioned to captivity (PERMANOVA, *F*_3,65_ *=* 3.5*, P =* 0.004; Fig. 1b). The influence of transitioning to captivity on microbial community composition is particularly apparent along the first principal component (PC1; Fig. 1b, Pearson’s *r*: 0.46). By contrast, richness and evenness (Shannon) and phylogenetic diversity (Faith’s PD) remained relatively stable across the first year in captivity, with no significant differences observed across time periods (Shannon, *F*_3,51_ *=* 2.0, *P =* 0.13; Fig. 1d; Faith’s PD, *F*_3,51_ = 0.58, *P* = 0.63). Interestingly, we did find wild F_0_ male pocket mice to have significantly lower Shannon diversity than F_0_ female pocket mice (*F*_1,26_ *=* 4.6, *P =* 0.04). By comparing the composition of the pocket mice’s gut microbiome across generations in captivity, we observed how dramatic this shift is for wild-born animals relative to subsequent captive-born generations (see PC1, Fig. 1c; Fig. S4a). However, significant compositional changes in subsequent captive-born generations are observed along PC2, with each generation being significantly different from the previous, excluding F_3_–F_4_ (PERMANOVA, F_0_–F_1_, pseudo-*F*_1,27_ *=* 36.6, *P =* 0.001; F_1_–F_2_, pseudo-*F*_1,30_ *=* 9.1, *P =* 0.006; F_2_–F_3_*,* pseudo-*F*_1,30_ *=* 5.6, *P =* 0.0004; F_3_–F_4_, pseudo-*F*_1,32_ *=* 1.9, *P =* 0.11; respectively; Fig. S4b). Interestingly, we observed the opposite relationship with dispersion (PERMDISP; pseudo-*F*_5,90_ *=* 2.5, *P =* 0.025). Across F_0_-wild to F_3_, we saw no significant difference in compositional variance across these generations (PERMDISP, all *P >* 0.29), but we observed differences in variance between both wild-born pocket mice and F_2_ generations compared to F_4_ (PERMDISP, F_0_-wild-F_4_, pseudo-*F*_1,31_ *=* 16.5, *P =* 0.002; F_0_-captive-F_4_, pseudo-*F*_1,26_ *=* 10.5, *P =* 0.009; F_2_–F_4_, pseudo-*F*_1,29_ *=* 11.2, *P =* 0.012, respectively). Taken together, compositional shifts that are occurring across F_0_–F_3_ in combination with similarly high levels of dispersion across generations prior to F_4_ suggest that the microbiome community composition stabilized after F_3_ (Fig. 1c; Fig. S1b). Despite the observed shifts in the pocket mouse gut microbiome composition across generations, there was no corresponding change in the level of Shannon diversity (*F*_5,121_ *=* 1.1, *P =* 0.38; Fig. 1e) or Faith’s PD (*F*_5,121_ *=* 1.0, *P =* 0.42).

### Microbial extirpation and replacement occur through several generations in captivity

**Fig 2:**
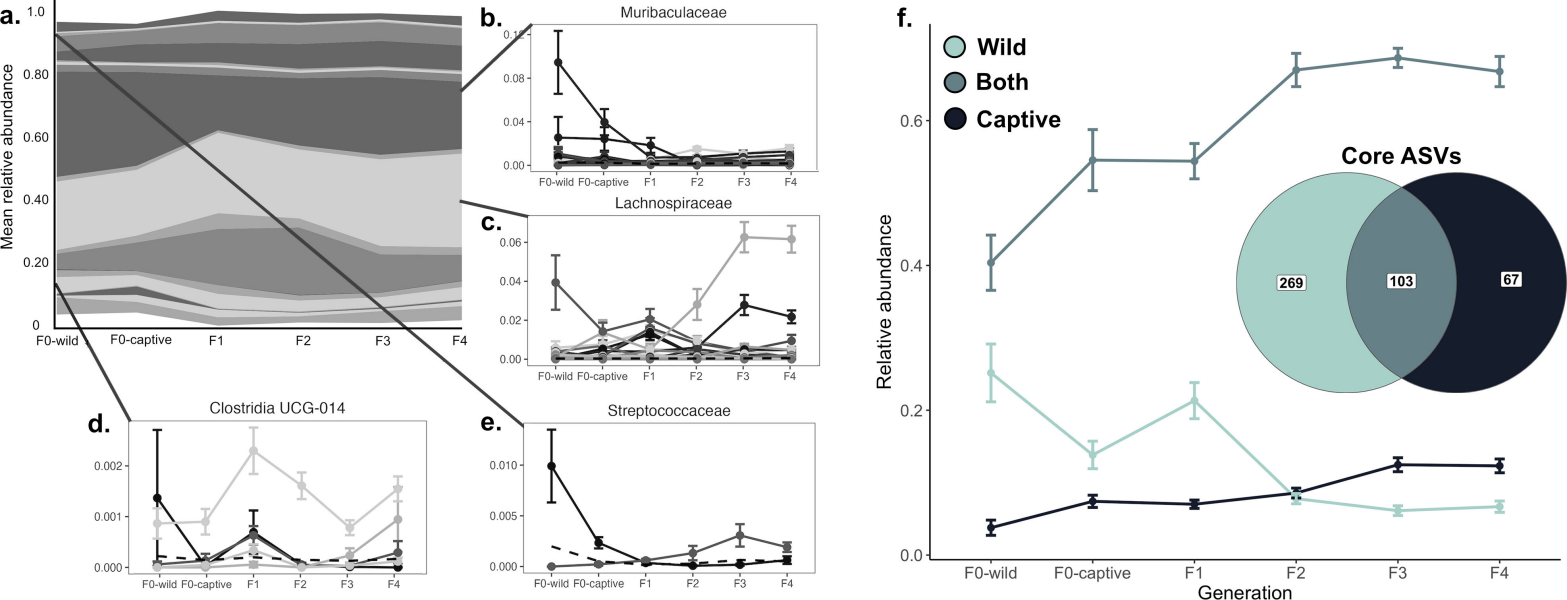
The mean relative abundance of core microbial families remained relatively constant across generations, while some individual ASVs within those families underwent extirpation and replacement. (**a**) Stream graph displaying the mean relative abundance of each family deemed core by an abundance-occupancy analysis across generations. The width of each bar illustrates the mean proportion of the core microbiome composed of that family in each generation. Panels **b–e** display ASVs that exhibited significant differences in mean relative abundance across generations, illustrating that while families remained constant, individual ASVs showed substantial flux. (**f**) The pocket mice’s core microbiome shifts to a new conformation—including different ASVs after several generations in captivity (inset Venn diagram). The ASVs considered core in wild animals (light blue) decrease in mean relative abundance and are replaced by ASVs that are core in captive animals (dark blue). Error bars represent standard error.

Across all pocket mice sampled, we found the most dominant bacterial phyla were Firmicutes (mean relative abundance = 62.2%), Bacteroidota (29.3%), Cyanobacteria (3.10%), Proteobacteria (2.10%), Actinobacteriota (1.70%), and Verrucomicrobiota (1.50%). At the family level, the dominant six taxa were classified as Lachnospiraceae (24.5%), Muribaculaceae (21.7%), Erysipelotrichaceae (15.3%), Rikenellaceae (7.00%), Ruminococcaceae (5.70%), and Clostridia UCG-014 (3.60%). Most ASVs that were shown to be differentially abundant across generations were members of microbial families that were present across all generations. The proportion of these core bacterial families remained relatively constant across generations (Fig. 2a), while ASVs within those families underwent an extirpation and replacement process (Fig. 2b through e). Some ASVs that dominated the community in wild animals were almost completely lost in F_1+_ individuals. For example, a Lachnospiraceae ASV and a Streptococcaceae ASV were both replaced by novel ASVs within the same bacterial family (Fig. 2c and e, respectively). In other cases, one dominant ASV in wild pocket mice, a member of the Muribaculaceae, was replaced by several lower abundance taxa from different unclassified genera (Fig. 2b; Fig. S6), while multiple ASVs within the Clostridia family changed across generations (Fig. 2d). At the genus level, the relative abundance of *Bacteroides* decreased significantly from F_0_ to F_1_, while the relative abundance of *Peptococcus* increased (Fig. S6). Several unknown genera in the orders Clostridia and Gastranaerophilales and the class Bacilli increased in abundance at F_1_, before decreasing to stable relative abundances.

While the core families in the pocket mouse gut microbiota remained constant, the ASVs considered “core” to a “wild” (F_0_-wild) versus “captive” pocket mouse (F_3+_) only partially overlapped (Fig. 2f). In total, 372 ASVs were considered core to the wild population at collection, of these, 269 ASVs (comprising ~25% of the total microbial community) were considered core microbiota to wild pocket mice only. In captivity, 170 ASVs were considered core, with 67 ASVs (comprising ~15% of the F_3+_ population’s gut microbiome) considered to be core microbiota within captive pocket mice only. However, 103 taxa that were identified as core in both wild and captive pocket mice made up 40%–65% of the microbial community, respectively. Many ASVs that contributed to the core microbiota of wild pocket mice were lost during the transition to captivity, with crossover between the two core groupings occurring in the F_2_ generation (Fig. 2f). This shift to a novel core community corresponds to the stabilization observed in the gut microbial community composition.

**Fig 3:**
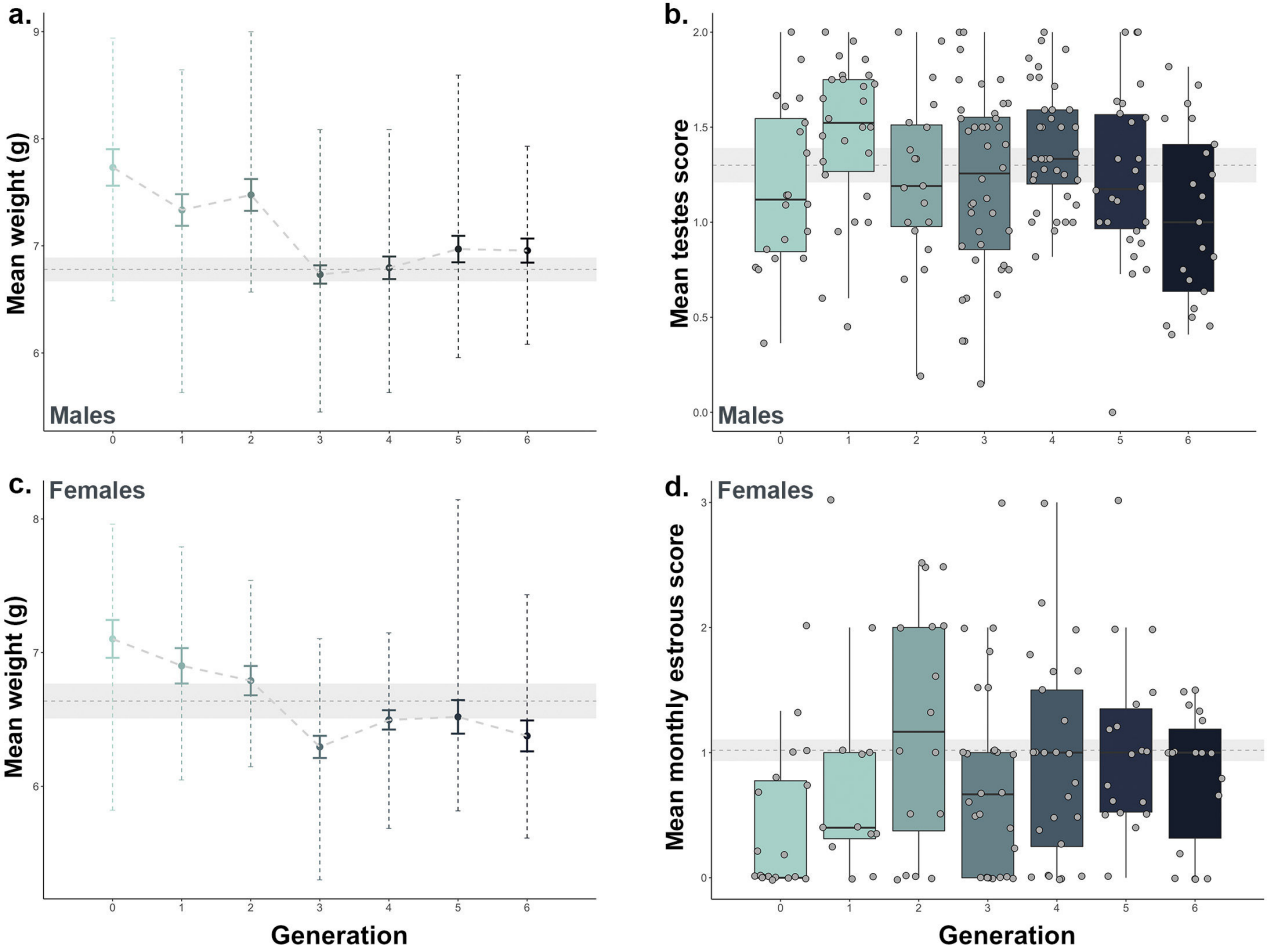
Fitness metrics change across pocket mice generations (F_0_ –F_6_). In the first generation, weights of both males (**a**) and females (**c**), male testes score (**b**), and female monthly estrous score (**d**) were compared to wild baseline (shown in gray, mean ± standard error baseline levels). Blue gradient indicates pocket mouse generation from F_0_ to F_6_ from light to dark. Error bars represent standard error.

**Fig 4:**
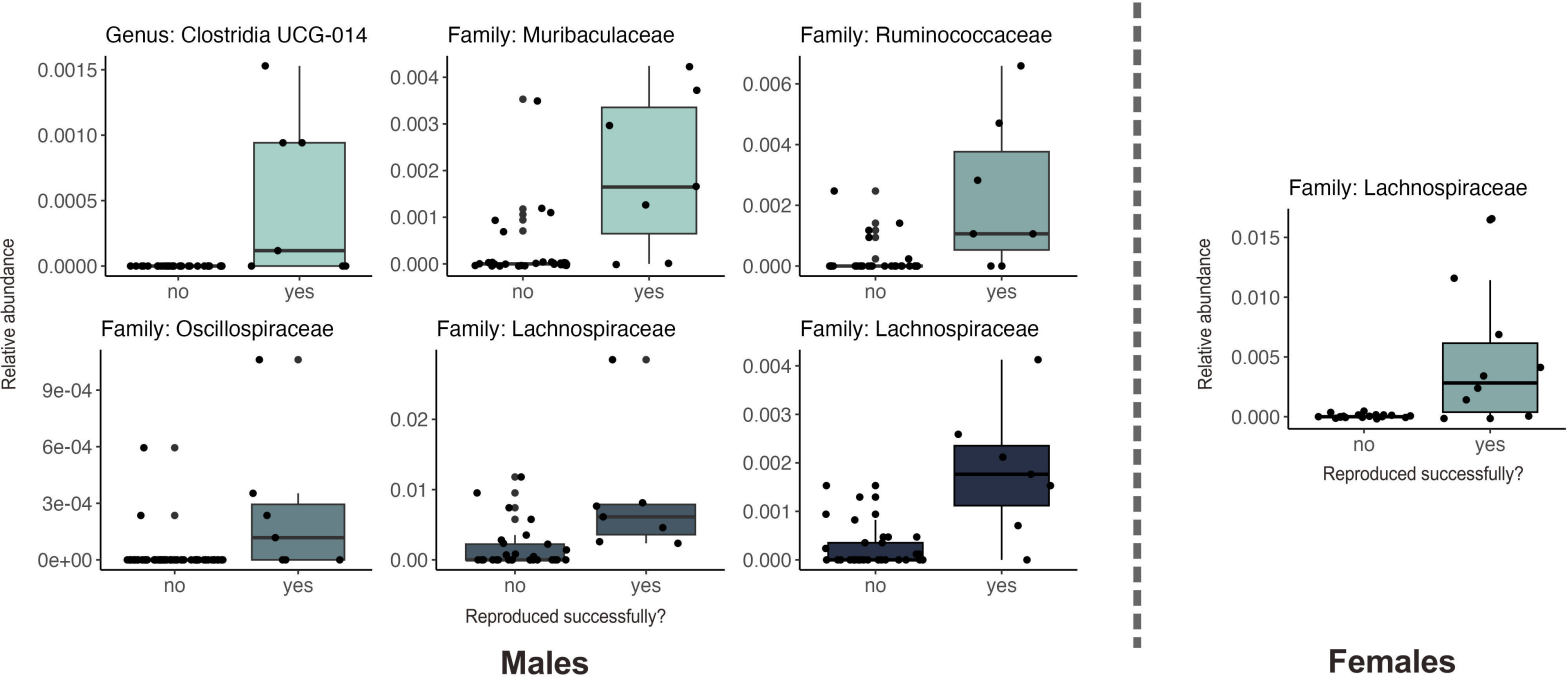
Relationship between microbial taxa and successful reproduction in the first full breeding season. Taxa associations are divided by males (left) and females (right). Each boxplot displays the relative abundance of each associated ASV, with the lowest taxonomy annotation available indicated in the plot title.

We examined weight and reproductive status as proxies for evaluating wildlife health across the entire *ex situ* population within the conservation breeding program from its inception (F_0_–F_6_) and used data collected *in situ* from extant populations as a baseline for comparison. We observed a transition in mean weight across generations in captivity (Fig. 3a and c, Kruskal-Wallis *Χ^2^* = 32.515 (males), 28.493 (females); *P <* 0.001 for males and females) where both male and female F_0_ in captivity have a significantly higher weight which declines and approaches the wild baseline after F_3_ and beyond (Wilcoxon *P <* 0.01 for F_0_ vs F_3_ comparison in both males and females). This shift toward the wild baseline is also observed in the reproductive metrics of male pocket mice transitioning to captivity (Fig. 3b, Kruskal-Wallis *Χ^2^* = 16.489, *P =* 0.01). Although not significant after correcting for multiple corrections, F_0_ and F_1_ females have fewer estrous cycles per month (Fig. 3d, Kruskal-Wallis *Χ^2^* = 12.304, *P =* 0.056), and estrous cycling increases toward the wild baseline, stabilizing in F_4_ individuals and beyond. This stabilization pattern mirrors what we observed with the microbiome data. Both pocket mice fitness and microbiome measures exhibit a clear period of flux through F_2_, after which point these metrics stabilize. Pocket mice adopt a novel, but stable microbial community in F_3_ and beyond, while reproductive metrics stabilize close to the wild baseline. Moreover, the relative abundance of some ASVs predicted pocket mouse reproductive fitness (Fig. 4, all *q*-values for reproduction <0.05). Despite the substantial flux we observed in the gut microbiome, these taxa did not change significantly from generation F_0_ through generation F_4_ (all *q*-values for generational differences >0.05).

## DISCUSSION

Rapid environmental change can impact animals in myriad ways, including through altering host-associated microbial communities (72). Captivity represents one of the most dramatic shifts an animal can experience, often producing strong physiological and fitness effects (8). Previous studies have documented differences between wild and captive microbiomes, but the dynamics of these changes within and across generations are not well described (23). Moreover, whether such changes have resultant effects on host fitness is unclear. Here, we tracked microbiome composition and physiological metrics of Pacific pocket mice as they transitioned to captivity within their first year and across five generations. We found that (i) the microbiome assumes a novel stable state alongside stabilized physiological metrics after three generations in captivity, (ii) microbial families remain constant despite extirpation and replacement of ASVs, and (iii) microbiome composition is related to reproductive success. These findings highlight the joint responses of hosts and their microbiota to rapid environmental change.

As pocket mice transitioned to captivity, their gut microbiome community composition shifted significantly, both within the first generation and through generation F_2_, after which no further significant changes occurred, indicating that a “captive” microbiome may have emerged in F_3+_ animals. Yet, these significant changes to microbiome community composition occurred despite no significant changes in alpha diversity. Reduction in alpha diversity has been proposed as a hallmark of captivity when compared to wild populations (23, 73), though this observation is not consistent in all species, and emerging evidence suggests that responses to captivity may be idiosyncratic among taxa and not a biologically meaningful indicator (74, 75). With repeat sampling, we observed a reduction in dispersion in the gut microbiota, indicating that while the individual-level alpha diversity of the microbiota does not change, the population-level variation is reduced in captive pocket mice. Pocket mice in our *ex situ* population receive native seed enrichment and live in a facility with skylights that allow for natural photoperiod and enclosures that allow for the retention of many of their natural behaviors, such as burrowing in sandy-soil substrates and living solitarily but with visual and olfactory conspecific cues. It is possible that one reason we may not see a significant loss of richness is through this successful mimicking of their wild diet and habitats (76, 77). Regardless, as alpha diversity remains constant, shifts in community composition occur due to changes in the relative abundance of taxa or the even extirpation and replacement of individual microbial ASVs.

Our study revealed that the mean relative abundances of core microbial families in pocket mice stayed relatively constant across generations, indicating that these families may serve important health and fitness functions for the species. These included taxa that are typical members of the mouse (*Mus* spp.) microbiome, including Muribaculaceae, which has been linked to complex carbohydrate utilization (78–80); Lachnospiraceae, a gut symbiont involved in nutrient acquisition and energy production (81, 82); Streptococcaceae, which are linked to memory and learning (79, 83); and Clostridia, which are associated with host metabolic homeostasis (84). However, we observed a significant change in the relative abundance of many different ASVs and several key genera within these core microbial families. For example, we noted a decrease in the abundance of a genus in the family Lachnospiraceae within the *Eubacterium ventriosum* group paired with an increase of a genus in the family Lachnospiraceae within the *Eubacterium xylanophilum* group. This shift might represent a transition from microbes adapted to wild, complex plant polysaccharides toward those adapted to xylan-rich seeds like sunflower seeds, millet, oat groats, and canary seed, which were fed in higher abundances in captivity to the pocket mice in our study (85). Such extirpation and replacement of ASVs and genera within core families may represent functional redundancy, buffering fitness impacts despite taxonomic turnover. Nonetheless, the limited taxonomic resolution for many ASVs highlights the need for improved annotations and functional studies to clarify microbial roles in pocket mouse health.

To understand if these extirpation and replacement dynamics led to a novel captive core community, we compared the ASV-level core microbiota of wild pocket mice to that of pocket mice in generations F_3_ and F_4_ (after the microbial community had stabilized, referred to as the “captive” microbiome). While many core ASVs overlapped, we observed a loss of many wild-core ASVs alongside an increase in captive-core ASVs, indicating that there are conserved microbiota, as well as microbiota that may be more substantially influenced by the environment, leading to population-level differentiation. It is unclear what the exact source of these novel ASVs is. While diet is certainly a driver of the changes in the gut microbiome in captivity, interaction with humans, the built environment, or a combination of these factors may also contribute novel taxa (86). Taken together, the shift between a wild and captive core microbiota but not a change in their proportion in the overall microbiome, coupled with high intra-generational compositional variance during the transition between wild and captive microbiota, may indicate a dynamic change between alternative stable states in the gut microbiome (87).

We documented a substantial deviation from the wild baseline values for both weight and estrous cycling in early generations in captivity. However, by generation F_2_–F_3_, we observed a transition back to the wild baseline for these metrics, followed by a stabilization. This occurs along the same timeline as the stabilization observed in the gut microbiota. Not only do we find similar stabilization trends between microbiome and host fitness metrics, but we also found the presence of several microbes to be predictive of successful male and female reproduction in their first year, including three Lachnospiraceae ASVs. Several studies have shown links between the gut microbiome and reproductive physiology, including microbial transformation of endogenous hormones or their endocrine-disrupting chemical mimics, such as phytoestrogens (13, 30). More specifically, members of Lachnospiraceae have been associated with increased fertility in captive female southern white rhinoceros (30) and reproductive hormone cycling, including a negative correlation to fecal progesterone metabolites in captive female black rhinoceros (88). As progesterone concentrations are typically low in early follicular and pre-ovulatory phases, while estrogen is high (89), an inverse relationship may also be inferred. In other words, a negative correlation of Lachnospiraceae ASVs with progesterone may equate to a positive correlation to pre-ovulatory phases prior to estrus.

Taken together, the simultaneous changes in gut microbiota and reproductive measures and correlations between specific taxa and reproductive success provide further evidence of a bidirectional connection between the gut microbiome and reproductive physiology. Specifically, the transition to captivity may induce changes in the gut microbial community, leading to an unstable transition state that might negatively affect host reproduction (87). After several generations in captivity, a novel, but healthy, alternative stable microbiome state may emerge that is suitable for the present host environment (17, 18, 90, 91). Close monitoring of populations through the first few generations in captivity and after reintroduction is therefore important, given that the host and microbiome both experience substantial disturbance that persists through this period. While our results provide evidence of a bidirectional interaction between reproduction and microbiota, it remains possible that the gut microbiome is simply an indicator of physiological stress, which responds, alongside many other aspects of host physiology, to rapid environmental change. Future work aimed to directly manipulate specific microbiota and measure host responses would help validate this relationship and further enable directed microbial restoration of *ex situ* pocket mice to improve the fitness of individuals slated for reintroduction to the wild.

While our results provide substantial insight into the dynamics of the gut microbiome as it responds to captivity within one and across multiple generations, several of our results should be interpreted with caution. First, due to the small population sizes of wild pocket mice, our conservation breeding program is composed of a small number of host genotypes (92), and reproduction was low across several early generations. Therefore, it remains difficult to disentangle the effects of host genetic variation versus environment on the gut microbiome and reproductive metrics in our population. This is an area of active debate (93, 94), and future work should attempt to parse out the effects of genetic variation and the gut microbiome on host response to environmental change using data collected in conservation breeding programs. Regardless, it is likely that both host genetic variation and gut microbiome composition play a role in host health and fitness and may need to be considered together for future conservation management decisions (95).

As anthropogenic activity continues to alter ecosystems across the globe and threaten the persistence of many species, human interventions like conservation breeding programs are becoming essential for species recovery. Our results demonstrate that the gut microbiome can respond to the pressures of captivity and also impact host fitness (28, 30, 96, 97). Thus, understanding how the gut microbiome will impact the success of these programs—both upon entering captivity and upon reintroduction—will influence recovery of at-risk species. More broadly, our study elucidates the effects of environmental change on organisms and their gut microbiota. We illustrate that environmental perturbations can result in an altered, highly variable gut microbiome that persists for multiple generations. However, eventually, the gut microbiome can stabilize alongside host metrics in the novel environment, providing hope for the long-term success of such conservation programs. How transitions between stable states of the gut microbiome, and their tipping points, might be directly linked with host fitness remains an open question. Regardless, gut microbiota appear to be an important piece of the puzzle when understanding how animals may respond to rapid changes in their environment, with significant implications for the persistence and recovery of endangered species.

## Data Availability

Raw sequences and corresponding metadata are available in the NCBI Sequencing Read Archive BioProject (PRJNA1031682). All other relevant data, protocols, and scripts, including all statistical models used for analysis, are available in a public GitHub repository (https://github.com/clw224/2023-Williams-PPM-WildtoCaptive/).

## SUPPLEMENTAL MATERIAL

Supplemental Material mbio.03516-25-s0001.docx Supplemental figures and Table S1 caption.

Table S1 mbio.03516-25-s0002.pdf Trimmed data set.

ASM does not own the copyrights to Supplemental Material that may be linked to, or accessed through, an article. The authors have granted ASM a non-exclusive, world-wide license to publish the Supplemental Material files. Please contact the corresponding author directly for reuse.
