# Supplementary material for: Captivity drives multi-generational shifts in the gut microbiome that mirror changing animal fitness
Source: mBio. 2026 Jan 23;17(2):e03516-25. doi: 10.1128/mbio.03516-25 (PMC12892941; doi:10.1128/mbio.03516-25)
Supplement: Supplemental Material — Supplemental figures and Table S1 caption. [file mbio.03516-25-s0001.docx]

**Supplemental information**

**
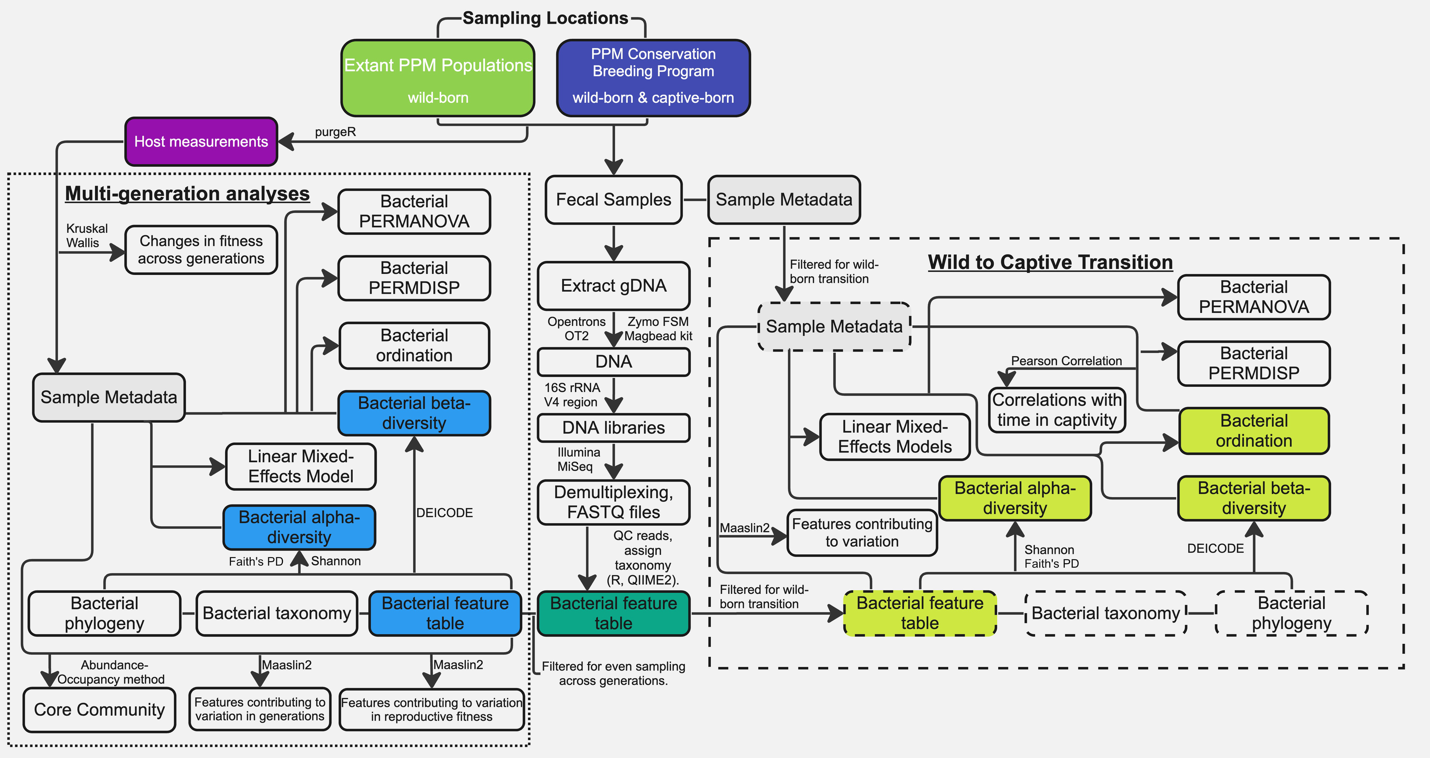
**

**Figure S1.** Visual workflow of all methods and analyses conducted for this manuscript.


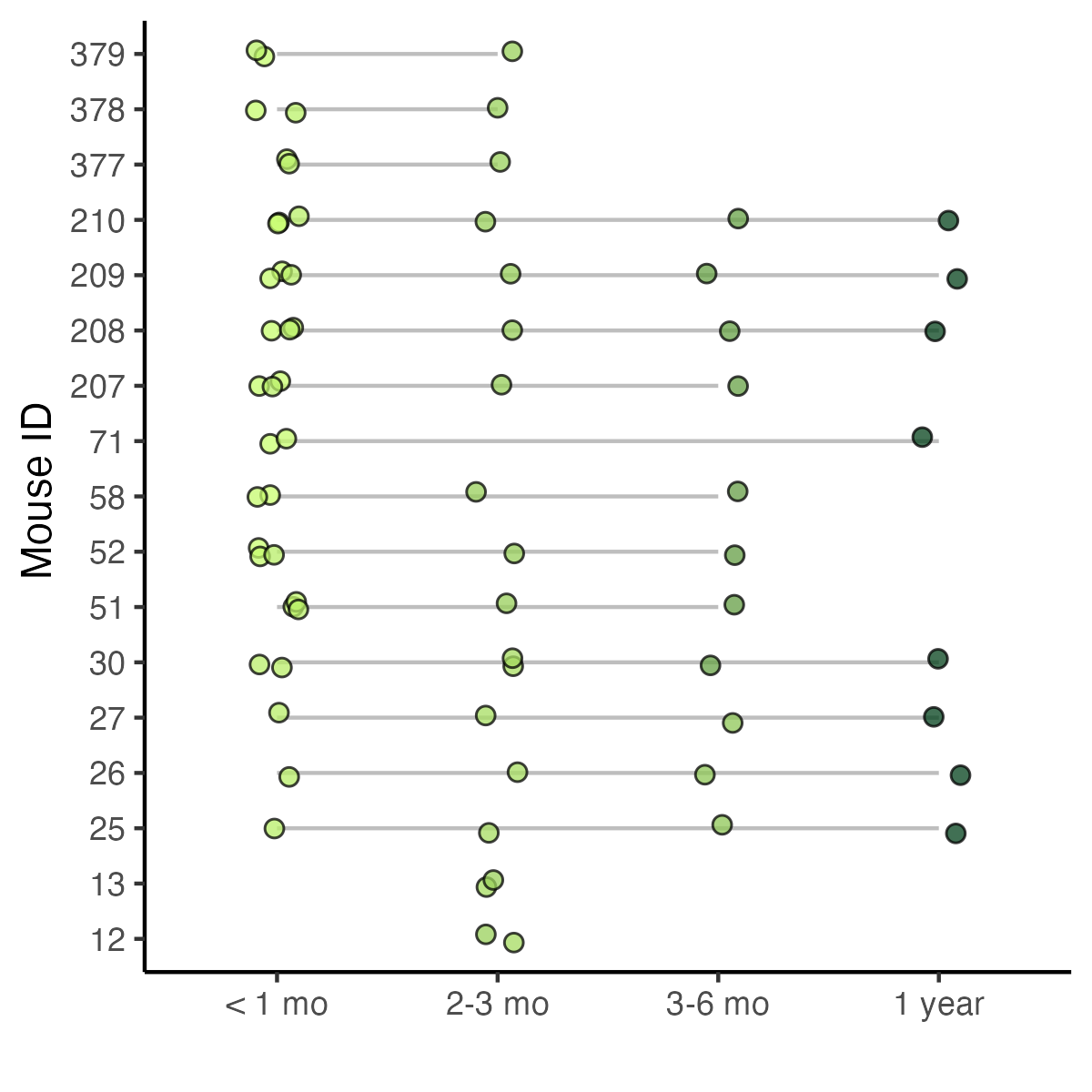


**Figure S2.** Multiple samples were analyzed for seventeen Pacific pocket mice founders over a period of 369 days after collected in the wild and brought into a captive breeding program.

**
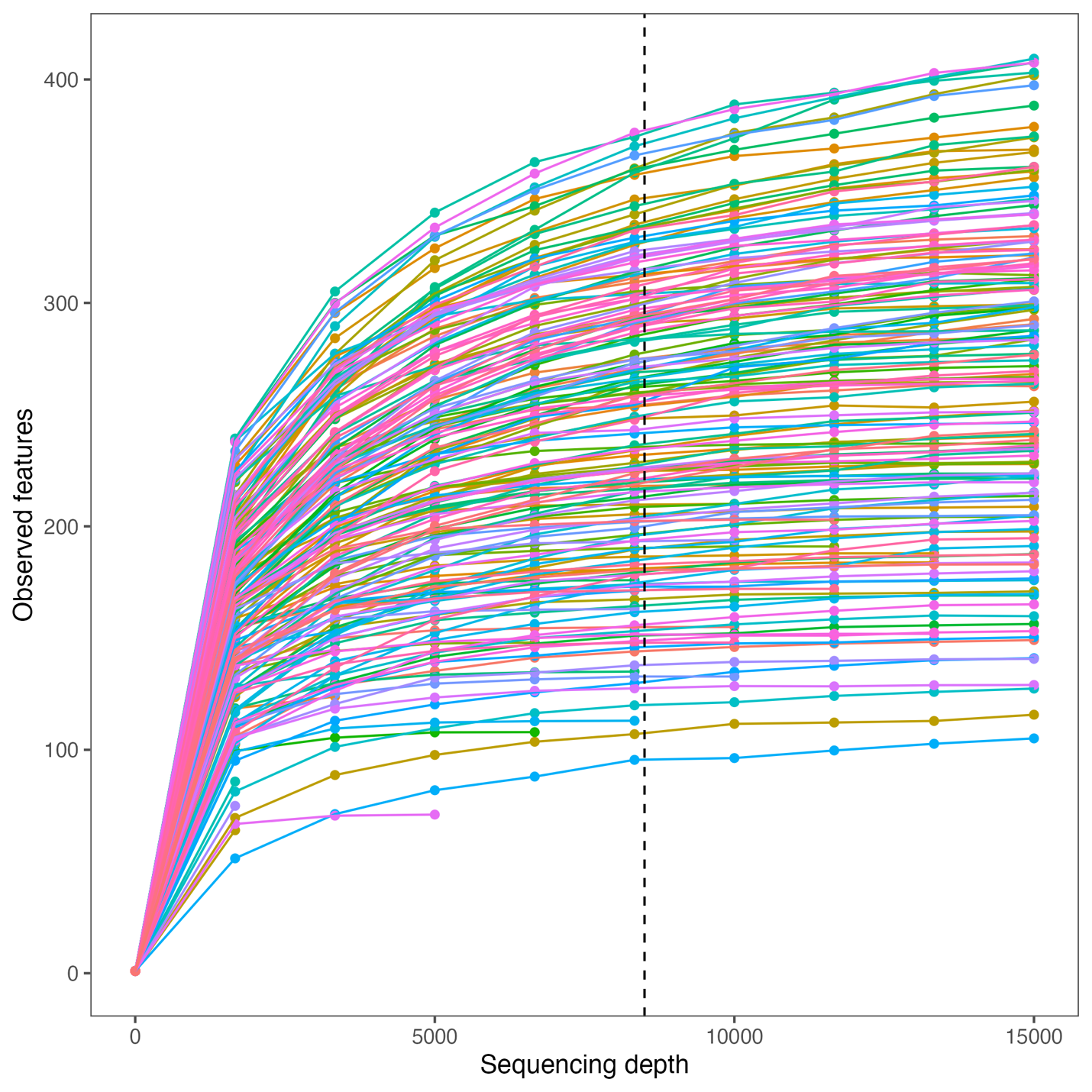
**

**Figure S3.** Rarefaction based on observed ASVs for all pocket mouse samples in dataset.

***
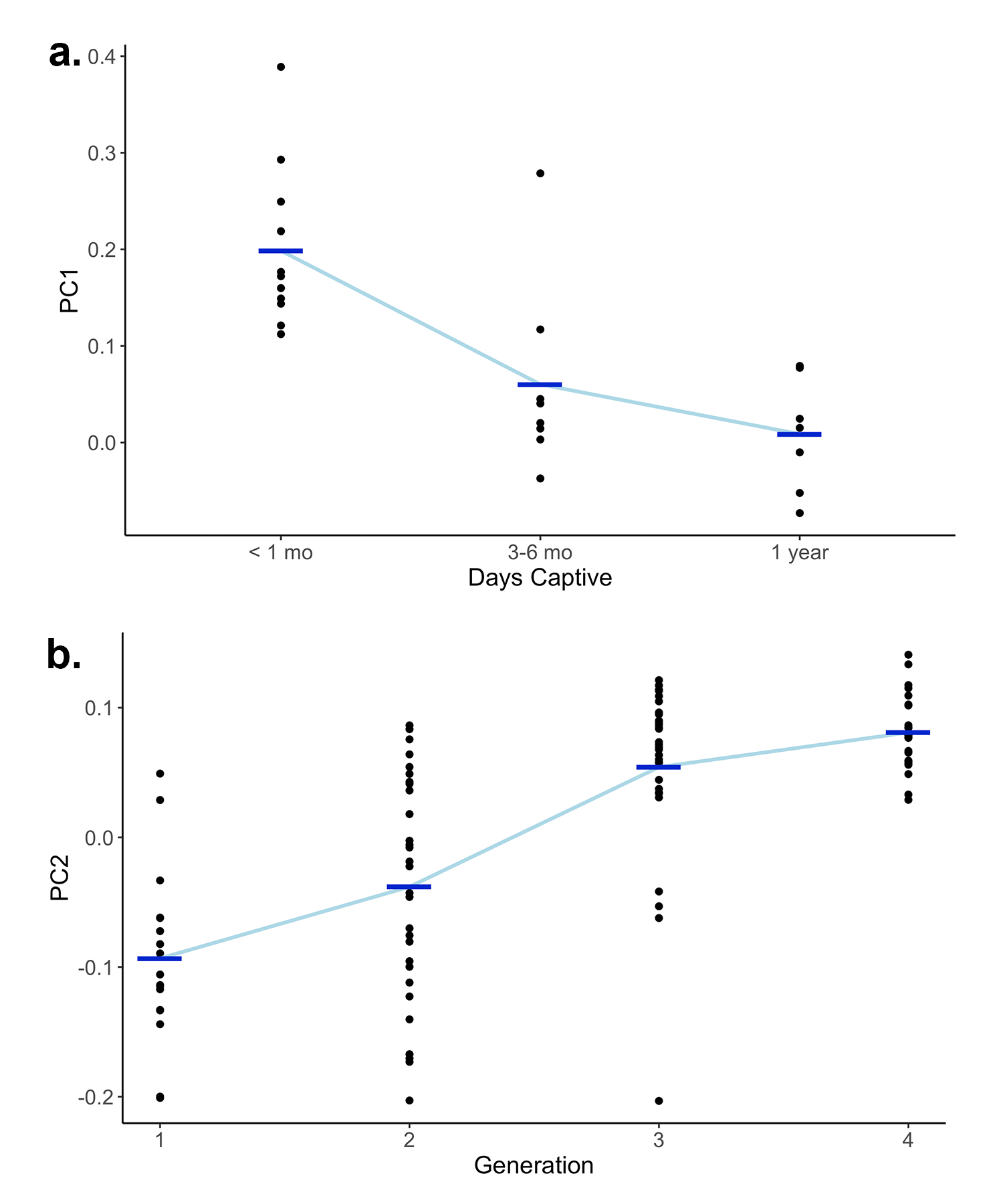
***

**Figure S4.** Different principal component axes (extracted from the ordination shown in Figure 1c) correspond to different time scales of change. Change along PC1 (a) is primarily driven by the number of days captive for F_0_, while change along PC2 (b) is primarily related to generation in captivity (F_1-4_).


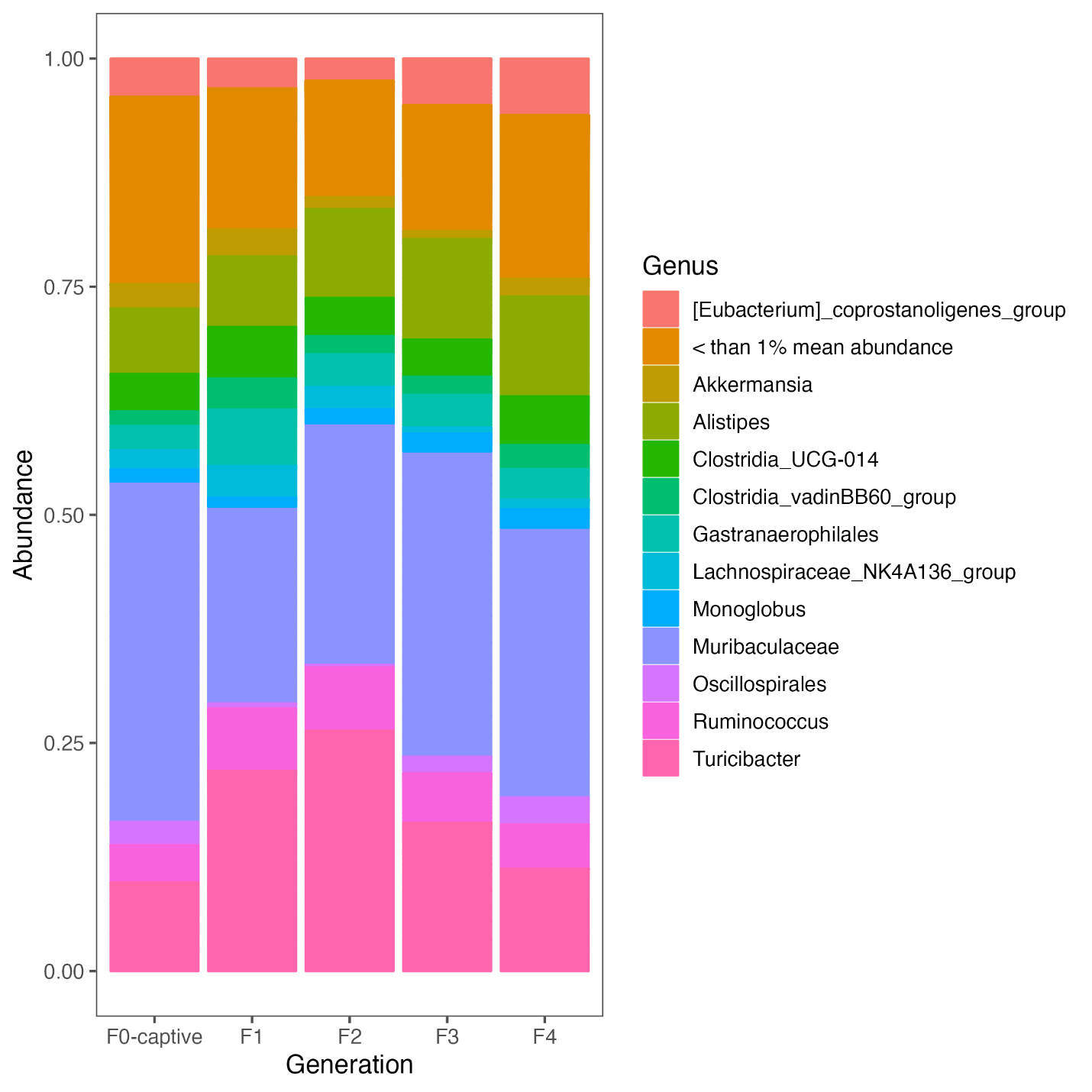


**Figure S5.** The mean relative abundance of bacterial genera over multiple generations.

**
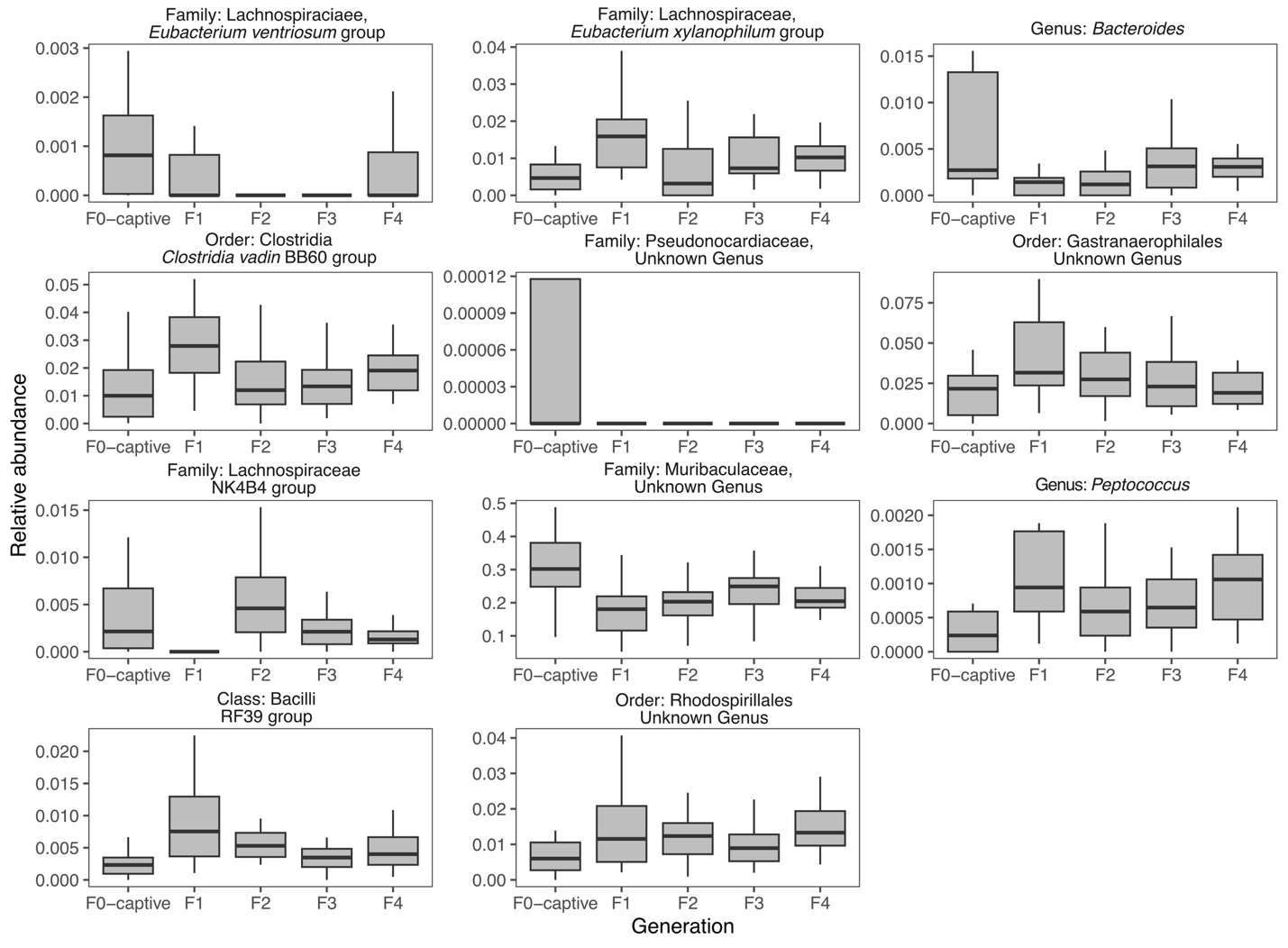
**

**Figure S6.** Generation in captivity alters the relative abundance of several genera, with the lowest taxonomy annotation available indicated in the plot title.

**Table S1.** A randomly trimmed dataset provides a balance of sample observations across generations (See Supplementary for data file).
