# Supplementary material for: Captivity drives multi-generational shifts in the gut microbiome that mirror changing animal fitness
Source: mBio. 2026 Jan 23;17(2):e03516-25. doi: 10.1128/mbio.03516-25 (PMC12892941; doi:10.1128/mbio.03516-25)
Supplement: Table S1 — Trimmed data set. [file mbio.03516-25-s0002.pdf]

Table S1. Trimmed, even dataset

Sample_ID	Mouse_ID	Generation	Sex
PPM-025-captive	25	F0	Male
PPM-026-captive	26	F0	Female
PPM-027-captive	27	F0	Male
PPM-030-captive	30	F0	Female
PPM-051-captive	51	F0	Male
PPM-051-wild	51	F0	Male
PPM-052-captive	52	F0	Female
PPM-052-wild	52	F0	Female
PPM-058-captive	58	F0	Male
PPM-058-wild	58	F0	Male
PPM-071-captive	71	F0	Female
PPM-071-wild	71	F0	Female
PPM-207-captive	207	F0	Male
PPM-207-wild	207	F0	Male
PPM-208-captive	208	F0	Female
PPM-208-wild	208	F0	Female
PPM-209-captive	209	F0	Male
PPM-209-wild	209	F0	Male
PPM-210-captive	210	F0	Female
PPM-210-wild	210	F0	Female
PPM-346-captive	346	F0	Female
PPM-347-captive	347	F0	Male
PPM-348-captive	348	F0	Female
PPM-349-captive	349	F0	Male
PPM-377-wild	377	F0	Female
PPM-378-wild	378	F0	Female
PPM-379-wild	379	F0	Male
PPM-031	31	F1	Male
PPM-032	32	F1	Female
PPM-033	33	F1	Female
PPM-034	34	F1	Female
PPM-035	35	F1	Male
PPM-036	36	F1	Male
PPM-037	37	F1	Male
PPM-038	38	F1	Male
PPM-039	39	F1	Female
PPM-040	40	F1	Male
PPM-041	41	F1	Male

PPM-042	42	F1	Male
PPM-043	43	F1	Female
PPM-044	44	F1	Male
PPM-046	46	F1	Male
PPM-113	113	F1	Female
PPM-231	231	F1	Female
PPM-062	62	F2	Male
PPM-063	63	F2	Male
PPM-064	64	F2	Female
PPM-065	65	F2	Female
PPM-066	66	F2	Male
PPM-067	67	F2	Male
PPM-068	68	F2	Male
PPM-069	69	F2	Female
PPM-070	70	F2	Female
PPM-078	78	F2	Male
PPM-079	79	F2	Female
PPM-080	80	F2	Male
PPM-085	85	F2	Male
PPM-086	86	F2	Female
PPM-087	87	F2	Female
PPM-088	88	F2	Female
PPM-089	89	F2	Male
PPM-090	90	F2	Female
PPM-091	91	F2	Male
PPM-092	92	F2	Female
PPM-093	93	F2	Female
PPM-096	96	F2	Male
PPM-097	97	F2	Male
PPM-099	99	F2	Female
PPM-119	119	F2	Male
PPM-135	135	F2	Female
PPM-136	136	F2	Male
PPM-137	137	F2	Male
PPM-216	216	F2	Female
PPM-122	122	F3	Female
PPM-131	131	F3	Male
PPM-132	132	F3	Female
PPM-134	134	F3	Male
PPM-141	141	F3	Male

PPM-142	142	F3	Female
PPM-143	143	F3	Male
PPM-144	144	F3	Male
PPM-145	145	F3	Female
PPM-156	156	F3	Female
PPM-157	157	F3	Male
PPM-162	162	F3	Female
PPM-163	163	F3	Male
PPM-164	164	F3	Male
PPM-165	165	F3	Female
PPM-166	166	F3	Male
PPM-167	167	F3	Female
PPM-168	168	F3	Male
PPM-170	170	F3	Female
PPM-171	171	F3	Male
PPM-172	172	F3	Female
PPM-173	173	F3	Male
PPM-174	174	F3	Female
PPM-175	175	F3	Female
PPM-196	196	F3	Male
PPM-197	197	F3	Female
PPM-198	198	F3	Male
PPM-199	199	F3	Female
PPM-245	245	F3	Male
PPM-247	247	F3	Female
PPM-248	248	F3	Female
PPM-154	154	F4	Female
PPM-155	155	F4	Male
PPM-158	158	F4	Female
PPM-159	159	F4	Female
PPM-160	160	F4	Male
PPM-161	161	F4	Male
PPM-188	188	F4	Male
PPM-189	189	F4	Male
PPM-190	190	F4	Male
PPM-191	191	F4	Male
PPM-200	200	F4	Male
PPM-201	201	F4	Male
PPM-202	202	F4	Male
PPM-203	203	F4	Female

PPM-204	204	F4	Male
PPM-205	205	F4	Male
PPM-206	206	F4	Female
PPM-218	218	F4	Female
PPM-220	220	F4	Female
PPM-224	224	F4	Male
PPM-225	225	F4	Female
PPM-227	227	F4	Female
PPM-257	257	F4	Male
PPM-260	260	F4	Male
